# An R toolbox for score-based measurement invariance tests in IRT models

**DOI:** 10.3758/s13428-021-01689-0

**Published:** 2021-12-16

**Authors:** Lennart Schneider, Carolin Strobl, Achim Zeileis, Rudolf Debelak

**Affiliations:** 1 University of Tübingen, Tübingen, Germany; 2 Present Address: Ludwig Maximilian University of Munich, Munich, Germany; 3 Department of Psychology, University of Zurich, Zurich, Switzerland; 4 Universität Innsbruck, Innsbruck, Austria; 5 Institute of Psychology, University of Leipzig, Neumarkt 9, 04109 Leipzig, Germany

**Keywords:** Differential item functioning, Item response theory, Software tutorial

## Abstract

The detection of differential item functioning (DIF) is a central topic in psychometrics and educational measurement. In the past few years, a new family of score-based tests of measurement invariance has been proposed, which allows the detection of DIF along arbitrary person covariates in a variety of item response theory (IRT) models. This paper illustrates the application of these tests within the R system for statistical computing, making them accessible to a broad range of users. This presentation also includes IRT models for which these tests have not previously been investigated, such as the generalized partial credit model. The paper has three goals: First, we review the ideas behind score-based tests of measurement invariance. Second, we describe the implementation of these tests within the R system for statistical computing, which is based on the interaction of the R packages *mirt*, *psychotools* and *strucchange*. Third, we illustrate the application of this software and the interpretation of its output in two empirical datasets. The complete R code for reproducing our results is reported in the paper.

## Introduction

A property of psychological and educational tests that is desirable in many applications is that test takers of equal ability show the same probability of giving correct or positive responses to the individual items, and that this probability does not depend on other variables. This property is typically termed measurement invariance (Millsap, 2011). On a conceptual level, an absence of measurement invariance can indicate that a test measures irrelevant characteristics of the respondents and that its fairness is violated (Camilli, 2006). Many authors and international testing standards thus emphasize the importance of checking the presence of differential item functioning (DIF; Holland & Wainer, 1993) in psychological measurement (American Educational Research Association, 2014; Borsboom, 2006). We illustrate this problem by a typical example, which we will revisit later on in the paper:

Consider a questionnaire for the assessment of verbal aggression, as presented in the Verbal Aggression dataset that was described in the book of De Boeck and Wilson (2004) and the paper of De Boeck et al., (2011). An example item is “A bus fails to stop for me. I would want to curse.”. Item response theory (IRT) models can be used to describe the interaction of test takers and test items in such a scenario (De Boeck et al., 2004, 2011). Here, DIF corresponds to a situation where respondents from various subgroups that have the same level of verbal aggression differ with regard to their tendency to agree to items from this test. Often these subgroups of respondents can be described by combinations of person characteristics, which are termed covariates in the following.

Over the last decades, various tests for the detection of DIF have been proposed. Among the suggested methods, several families of tests can be discerned. Traditional approaches are based on the comparison of predefined groups of respondents (for an overview, see Magis et al.,, 2010). For this purpose, a reference group and one or more focal groups are defined before the analysis. A practical disadvantage of these traditional approaches is that it remains unclear how to address the problem of detecting DIF effects if no natural groups are available. This can occur, for instance, if the respondents differ with regard to continuous covariates such as age.

To address this problem, several alternative approaches were proposed to assess measurement invariance with respect to non-categorical person covariates. Liu et al., (2016) proposed a semiparametric IRT model and a permutation test which allows the detection of DIF effects with regard to a continuous covariate. Related approaches proposed IRT models which included person covariates in the item response functions (e.g., Moustaki, 2003). These models either require estimation of the form of the item response curve, or assume a specific relationship between the person covariate and the observed responses.

A second alternative approach is based on moderated factor analysis (Bauer, 2017, 2009, Molenaar, 2020). Here, person covariates are included in a factor model, which allows the modeling of changes of factor loadings and other model parameter with regard to person covariates. Moderation functions are used to describe the relationship between the observed person covariates and the other model parameters.

A third alternative approach is based on mixture distribution models. An important example is the mixed Rasch model (Rost, 1990). Here, a number of latent groups of respondents is assumed. Respondents from different groups differ with regard to the specific values of the item parameters. Whereas traditional mixture distribution models do not define a relationship between these latent groups and observed covariates, several authors have suggested extensions of mixture distribution models which include person covariates (Dai, 2013; Li et al., 2016; Tay et al., 2011). The resulting models allow an estimation of the relationship between observed covariates and the probability of individual respondents for being a member in a specific latent group.

In this paper, we present a family of score-based measurement invariance tests that can be used to check the presence of DIF with regard to observed continuous, ordinal and categorical covariates, such as age, educational level or gender. The resulting family of tests is very flexible and does not assume a specific relationship between the observed person covariates and possible DIF effects. These tests can be applied to a wide range of IRT models, both with conditional maximum likelihood (CML) and marginal maximum likelihood (MML) estimation methods (Baker and Kim, 2004). Table 1 gives an overview of the supported IRT models. Note that in this paper, we use IRT as a broad umbrella term for item response models that also includes Rasch-type models.

**Table 1:** Overview of supported IRT models

Model	Response type	Estimation	Model reference	Score-based tests published in
Rasch Model	dichotomous	CML	Rasch (1960)	Strobl et al., (2015)
1PL	dichotomous	MML	Birnbaum (1968)	
2PL	dichotomous	MML	Birnbaum (1968)	Debelak and Strobl (2019)
3PL	dichotomous	MML	Birnbaum (1968)	Debelak and Strobl (2019)
3PLu	dichotomous	MML	Barton and Lord (1981)	
4PL	dichotomous	MML	Barton and Lord (1981)	
ideal point model	dichotomous	MML	Maydeu-Olivares et al., (2006)	
rating scale model	polytomous	CML	Andrich (1978)	Komboz et al., (2018)
partial credit model	polytomous	CML	Masters (1982)	Komboz et al., (2018)
generalized partial credit model	polytomous	MML	Muraki (1992)	
graded response model	polytomous	MML	Samejima (1969)	
nominal response model	polytomous	MML	Bock (1972)	
generalized graded unfolding model	polytomous	MML	Roberts et al., (2000)	
monotonic polynomial model	polytomous	MML	Falk and Cai (2016)	

Note. For all models that can be fitted via MML using the R package *mirt* (Chalmers, 2012), mixed itemtype models as well as multidimensional confirmatory and exploratory versions and multiple group versions of the models are supported. Moreover, arbitrary parameter constraints are possible. This allows for fitting, e.g., a partial credit model via MML

As can be seen from Table 1, the software presented in this paper allows the calculation of score-based tests for various IRT models for which these tests have not been described or derived before. In summary, this tutorial paper provides the following contributions: First, we review the ideas behind score-based tests of measurement invariance. Second, we present their implementation in the R system for statistical computing (R Core Team, 2019). Third, we illustrate the application of this software and the interpretation of its output by means of two empirical datasets. The audience of this paper includes substantive researchers who wish to apply these methods to their own datasets as well as psychometricians who are interested in their theoretical background.

The following section “A Conceptual and Formal Framework for Score-Based Measurement Invariance Tests” briefly describes the underlying framework of these tests. The section “The Implementation of Score-Based Measurement Invariance Tests within R” describes their implementation within R (R Core Team, 2019). In the section “Illustrations with Empirical Data”, we illustrate this methodology by means of two datasets in a tutorial style. This section includes an analysis of the Verbal Aggression dataset described by De Boeck and Wilson (2004) and De Boeck et al., (2011) as well as an analysis of the Generic Conspiracist Beliefs dataset (Brotherton et al., 2013; Open Source Psychometrics Project, 2016). We conclude our paper with a general discussion in the final section.

## A conceptual and formal framework for score-based measurement invariance tests

### An overview

The class of score-based tests considered in the following was originally developed in the econometrics literature as a method to test for instability (or “fluctuation”) in ordinary least squares (OLS) or maximum likelihood (ML) estimates (Andrews, 1993). They can also be applied to other types of M-estimators (beyond OLS and ML), which is why they are named M-fluctuation tests in some publications (Strobl et al., 2015; Zeileis and Hornik, 2007). In the field of psychometrics, they were first described for Bradley-Terry models (Strobl et al., 2011), factor analytical models (Merkle et al., (Merkle & Zeileis, 2013; Merkle et al., 2014)) and the dichotomous Rasch model (Strobl et al., 2015). Later works generalized this method to other models of item response theory (Debelak & Strobl, 2019; Komboz et al., 2018; Wang et al., 2018), including the application of these tests in a model-based recursive partitioning framework to detect subpopulations that show measurement invariance (Komboz et al., 2018; Strobl et al., 2015).

The family of score-based tests can be considered a generalization of the classical Lagrange multiplier test that was applied by Glas and colleagues to detect DIF effects in various IRT models when pre-defined groups are available (Glas, 1998, 1999, 2003). Another closely related approach for checking the invariance of individual model parameters in dynamic panel models that is based on regression analysis was recently proposed by Arnold et al., (2020), using ideas presented by Oberski (2013).

To present the formal ideas behind these tests, we start by considering an IRT model whose model parameters are summarized by a vector $\boldsymbol {\Psi } = ({\Psi }_{1}, \dots , {\Psi }_{P})$ and a matrix of observed responses *U*. We further denote the rows of *U*, which correspond to responses given by *N* individual respondents, by $(\boldsymbol {u}_{1}, \dots , \boldsymbol {u}_{N})$. It follows from the local independence assumption made in most IRT models that the log-likelihood $\ell (\boldsymbol {\Psi }; \boldsymbol {u}_{1}, \dots , \boldsymbol {u}_{N})$ of these models is a sum of case-wise contributions:

$$ \ell(\boldsymbol{\Psi}; \boldsymbol{u}_{1}, \dots, \boldsymbol{u}_{N}) = \sum\limits_{i=1}^{N} \ell(\boldsymbol{\Psi}; \boldsymbol{u}_{i}). $$

Score-based tests use the scores of the model parameters to assess their invariance. Here the score is the gradient, i.e., the vector of first partial derivatives of the log-likelihood with respect to a model parameter of interest. Using our notation, the score is thus given by:

$$ s(\boldsymbol{\Psi}; \boldsymbol{u}_{1}, \dots, \boldsymbol{u}_{N}) = \left( \frac{\partial \ell(\boldsymbol{\Psi}; \boldsymbol{u}_{1}, \dots, \boldsymbol{u}_{N})}{\partial{\kern1pt}{\Psi}_{1}}, \dots, \frac{\partial \ell(\boldsymbol{\Psi}; \boldsymbol{u}_{1}, \dots, \boldsymbol{u}_{N})}{\partial{\kern1pt}{\Psi}_{P}} \right)^{t}. $$

As was the case for the log-likelihood, we can present the score as a sum of independent, individual score contributions:

$$ s(\boldsymbol{\Psi}; \boldsymbol{u}_{1}, \dots, \boldsymbol{u}_{N}) = \sum\limits_{i = 1}^{N} s(\boldsymbol{\Psi}; \boldsymbol{u}_{i}), $$

where

$$ s(\boldsymbol{\Psi}; \boldsymbol{u}_{i}) = \left( \frac{\partial \ell(\boldsymbol{\Psi}; \boldsymbol{u}_{i})}{\partial{\kern1pt}{\Psi}_{1}}, \dots, \frac{\partial \ell(\boldsymbol{\Psi}; \boldsymbol{u}_{i})}{\partial{\kern1pt}{\Psi}_{P}} \right)^{t}. $$

If we replace **Ψ** by its maximum likelihood estimate $\hat {\boldsymbol {\Psi }}$, we directly obtain:

$$ s(\hat{\boldsymbol{\Psi}}; \boldsymbol{u}_{1}, \dots, \boldsymbol{u}_{N}) = \sum\limits_{i = 1}^{N} s(\hat{\boldsymbol{\Psi}}; \boldsymbol{u}_{i}) = \boldsymbol{0}. $$

As can be seen, the sum over all individual score contributions, evaluated at the maximum likelihood estimate, is ***0***, that is, a vector of zeros. The reason is that by definition the score is ***0*** at the maximum likelihood estimator, where the log-likelihood reaches its maximum. Under the null hypothesis that the model underlying the estimation is the true model and all parameters are stable, we can also determine the asymptotic distribution of cumulative sums of scores. If the parameters are not invariant, the sum of the individual score contributions is still ***0***, but unexpected deviations from this asymptotic distribution occur. The basic idea of the score-based invariance tests is to first sort all respondents with regard to a person covariate of interest. We denote the resulting ordered responses by $(\boldsymbol {u}_{(1)}, \dots , \boldsymbol {u}_{(N)})$. Now, we consider the following term for a value *t* between 0 an 1:

$$ B(t, \hat{\boldsymbol{\Psi}}) = \hat{I}^{-1/2} N^{-1/2} \sum\limits_{i = 1}^{\lfloor Nt \rfloor} s(\hat{\boldsymbol{\Psi}}; \boldsymbol{u}_{(i)}). $$

Here, ⌊⌋ is the floor function and $\hat {I}$ denotes a consistent estimate of the covariance matrix of the case-wise score contributions. Its inclusion leads to a standardization of $B(t, \hat {\boldsymbol {\Psi }})$.

If no DIF is present, the expected distribution of $s(\hat {\boldsymbol {\Psi }}; \boldsymbol {u}_{(i)})$ will be independent from the index (*i*), that is, the ordering with respect to the person covariate. It follows that the distribution of the cumulative sum $B(t, \hat {\boldsymbol {\Psi }})$ is known for sufficiently large samples if the IRT model is correctly specified and under the null hypothesis that no DIF effects are present. Mathematically, this follows from the functional central limit theorem, or Donsker’s theorem (Billingsley, 1995).

**Fig. 1:**
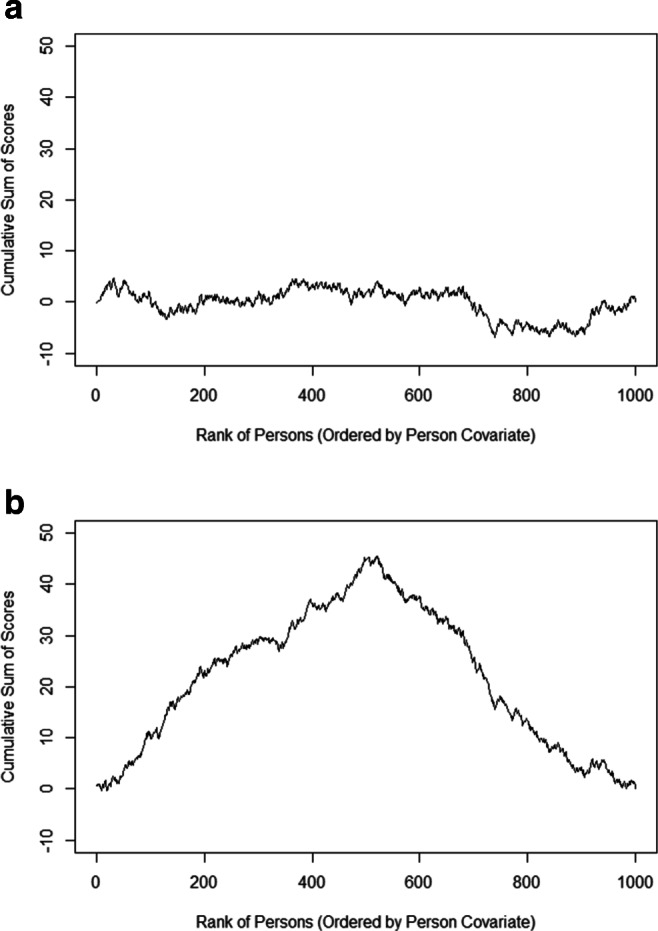
**a** The cumulative sum of scores if DIF is absent. The observed values fluctuate randomly around 0. **b** The cumulative sum of scores if DIF is present between the first and the second half of the sample. Instead of a random fluctuation, a typical pattern emerges

As an illustration, Fig. 1 gives two examples of the cumulative sum of scores for a single parameter in an IRT context when DIF is absent (top) or present (bottom). As can be seen, the cumulative sum fluctuates randomly around 0 if DIF is absent, but leads to a systematic pattern if DIF is present in a certain covariate. Although this illustration uses a continuous covariate, the same principle can be used for categorical and ordinal covariates.

The information on the invariance of the various model parameters can be summarized by test statistics, and the *p*-value under the null hypothesis of parameter invariance can be calculated. For computing the test statistic, we can use all available model parameters, focus on groups of model parameters or can even use only single model parameters. Conceptually, this allows the assessment of measurement invariance for the whole item set, groups of items or single items. In this tutorial, we will focus on invariance tests for the complete item set, but will also give an example for a test where invariance is checked for a single item.

Applications of score-based tests in IRT models share the following common steps for assessing the invariance of model parameters:

1. First, an appropriate model is chosen for describing the data and its parameters are estimated using a ML approach. In IRT models, this is typically done via CML or MML estimation. The user can be supported in this step by wrapper functions (see below).
2. Second, the case-wise score contributions are calculated and used for measuring the invariance of the parameter estimates with regard to some person covariate.
3. Third, this item-wise invariance information is summarized using a test statistic, either for the complete item set or a subset. The choice of test statistic typically depends on whether the person covariate that is used for the analysis is categorical, ordinal, or continuous.

**Fig. 2:**
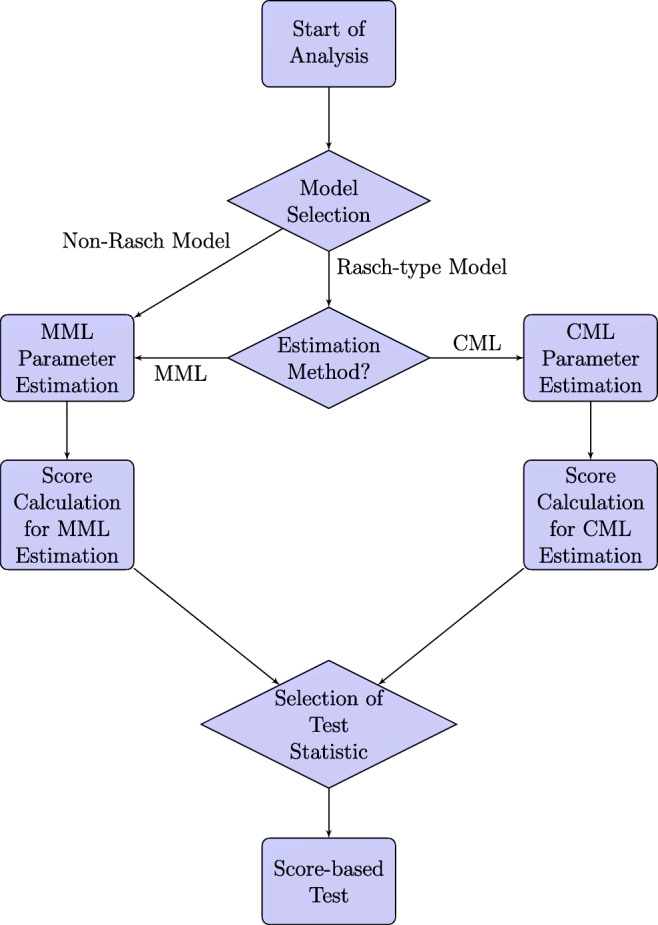
The workflow of the application of score-based measurement invariance tests for IRT models. Diamond-shaped nodes correspond to decisions made by the user, whereas rectangle-shaped nodes stand for steps in the resulting type of analysis

For IRT models, this leads to the workflow illustrated in Fig. 2.

In the following subsections, we will discuss these steps in more detail.

### Estimating the Item parameters of IRT models

Historically, numerous methods have been proposed for estimating IRT model parameters, but we focus here on two methods that are most widely used today: CML and MML estimation. CML estimation makes use of specific characteristics of Rasch-type models and can generally not be used to estimate the item parameters of more general IRT models, like the two-parametric logistic or 2PL model (Birnbaum, 1968). MML estimation can be used to estimate the item parameters in more general models, but makes specific assumptions on the person parameter distribution. For more detailed presentations, see Baker and Kim (2004) or Molenaar (1995). Once the item parameters have been estimated, the next step of the data analysis consists of the calculation of the individual score contributions.

### Determining the distributions of the individual score contributions

In IRT models, scores for individual item parameters can be described as sums over the respondent sample, with each summand being an individual score contribution. For an illustration, we consider the 2PL model in slope/intercept parametrization. Let *u*_*ij*_ denote the response of respondent *i* (*i* = 1,…,*N*) to item *j* (*j* = 1,…,*J*). In this model, the probability of a positive response of respondent *i* to item *j* depends on an item-specific slope parameter *a*_*j*_, an item-specific intercept parameter *d*_*j*_, and a respondent-specific ability parameter *𝜃*_*i*_ through the item response function:

$$ P_{ij} := P(u_{ij} = 1 | a_{j}, d_{j}, \theta_{i}) = \frac{\exp(a_{j} \theta_{i} + d_{j})}{1 + \exp(a_{j} \theta_{i} + d_{j})}. $$

As an example, the derivative of the marginal log-likelihood with regard to the slope parameter takes on the following form (Baker and Kim, 2004; Debelak & Strobl, 2019):

$$ \sum\limits_{i=1}^{N} \int (u_{ij} - P_{ij}) \cdot \theta_{i} \cdot P(\theta_{i}|\boldsymbol{u}_{i}, \boldsymbol{\Psi}) d \theta_{i}. $$

In this equation, *P*(*𝜃*_*i*_|***u***_*i*_,**Ψ**) depends on the model parameters, which we summarize as **Ψ**, and the responses given by respondent *i*, which we summarize as ***u***_*i*_. This equation shows again that the derivative can be presented as a sum of individual contributions of the *N* respondents. This observation also holds for the other item parameters. It is thus possible to calculate the individual score contributions for all respondents and all item parameters given the observed response data and the item parameter estimates.

### Testing measurement invariance with score-based tests

The presence of DIF leads to a systematic deviation of the observed distribution of the cumulative individual score contributions from the distribution that is expected when no DIF is present. It is therefore necessary to summarize the deviations found in all item parameters. Several test statistics have been proposed in the literature for this purpose. Here we provide a brief overview of some selected test statistics.

In the following tutorial, we confine ourselves to the *LM* test for *u* n*o* rdered covariates (denoted LMuo), the *max* imally-selected *LM* test across the levels of an *o* rdered covariate (denoted maxLMo), and the *max* imally-selected *LM* test for a continuous covariate (denoted maxLM). Another available option is the *d* ouble-*m* aximum test statistic (denoted DM), which is given by the maximum (over all respondents) of the maximum (over all item parameters) of the empirical cumulative process of the standardized individual score contributions (Merkle et al., 2013, 2014). The double-maximum statistic is designed for detecting DIF effects with regard to continuous covariates. More details regarding the tests and their construction are provided by Merkle and Zeileis (2013) and Merkle et al., (2014), who also point out that other types of test statistics – e.g., (weighted) double maximum tests or Cramér-von-Mises-type tests – can be easily computed. Wang et al., (2014) give a practical introduction.

All of these statistics can be used to summarize the information of the invariance of various item parameters. The user can decide to summarize this information for the complete item set, leading to a DIF test for all items. Alternatively, this information can be summarized for individual items or groups of items, leading to item-wise DIF tests and DIF tests for groups of items, respectively.

## The implementation of score-based measurement invariance tests within R

All functions needed for a DIF analysis with score-based tests are made available via three R packages, which are *psychotools*, *mirt* and *strucchange*. In this section, we will summarize their interaction and illustrate their application with example datasets.

### The interaction of the R packages

The application of score-based measurement invariance tests conceptually consists of three major steps, which are: 1. the selection and estimation of a suitable IRT model, 2. the calculation of the individual score contributions, 3. the selection of a suitable test statistic and the calculation of the corresponding *p*-value. These steps correspond to specific steps in the analysis (see again Fig. 2):

- First, an appropriate IRT model has to be chosen based on theoretical considerations and on the type of items. If a Rasch-type model is chosen, for instance, the user can choose the CML or MML method for estimating the item parameters. If the model is specified correctly, both approaches lead to comparable results. On a technical level, *psychotools* allows the estimation of the item parameters with the CML method, whereas *mirt* allows the estimation of the item parameters using the MML approach.
- Second, the scores and individual score contributions are calculated. This is typically not done explicitly by the user, but done implicitly during the analysis. Like in the first step, these score contributions are calculated by *psychotools* using the CML approach and by *mirt* using the MML approach.
- Third, the user has to select a test statistic. This step is necessary for the calculation of a *p*-value via *strucchange*.

In summary, *mirt* and *psychotools* are used for the item parameter estimation and the calculation of the corresponding scores. *psychotools* is used for these calculations in the CML approach, whereas *mirt* is used with the MML approach. Based on these results, *strucchange* can be used for the calculation of *p*-values.

### Important R functions

This section summarizes the most important functions of each R package on a conceptual level. We will present the functions in the order of the general workflow. Therefore, we will first address functions for the estimation of the item parameters, then functions for the score estimation, and functions for carrying out score-based measurement invariance tests. Finally, we highlight some additional functions of the *psychotools* package. For all functions we also briefly introduce important arguments of the functions. To inspect the full list of arguments, the respective R help pages should be consulted. We start with some basic functions for fitting IRT models:

- raschmodel, rsmodel, pcmodel: These are basic fitting functions of the *psychotools* package for the binary Rasch model (rasch, Rasch, 1960) and two polynomial Rasch-type models, namely the rating scale model (rsmodel, Andrich, 1978) and the partial credit model (pcmodel, Masters, 1982). All of these functions estimate the item parameters within the CML approach. These functions require at least the response matrix provided via the argument y.
- plmodel, gpcmodel: These functions are user-friendly wrapper functions in *psychotools* that allow the estimation of common IRT models within the MML approach relying on *mirt*. plmodel allows the estimation of a wide range of models for dichotomous items, like the 2PL, 3PL (Birnbaum, 1968) and 4PL (Barton and Lord, 1981) models. gpcmodel allows the estimation of the generalized partial credit model (GPCM; Muraki, 1992). Both plmodel and gpcmodel also allow for fitting Rasch-type models within the MML approach, i.e., restricting the slopes to be equal for all items. Both functions require the response matrix as argument y. Modeling group differences in the true person parameter distributions, which is also named impact (e.g. Wang et al.,, 2012), is possible by the optional impact argument. This argument accepts a vector that defines a group membership for each respondent and models impact effects between the resulting groups. plmodel additionally accepts a type argument, which defines the estimated IRT model (e.g., by default type = "2PL").
- *psychotools*’ extractor functions: One advantage of using the fitting functions of the *psychotools* package, including the wrapper functions, is that *psychotools* provides plenty of easy to use extractor functions for all kinds of IRT parameters, i.e., item difficulty parameters, item discrimination parameters, lower and upper asymptote parameters, threshold parameters and also person parameters (itempar, discrpar, guesspar, upperpar, threshpar and personpar). Moreover, *psychotools* provides unified inference and visualization tools for its supported models.
- sctest: This function is part of the *strucchange* package. Based on the individual score contributions, a person covariate and the selection of a test statistic (or functional), this function allows for the calculation of the test statistic and the associated *p*-value. This function is a generic function and in the scenarios described here, dispatches the sctest.default method, which has three main arguments: x is an estimated IRT model object, that is the return value of mirt, raschmodel or similar commands. order.by denotes the person covariate that is tested for DIF. functional allows the definition of the test statistic used for the score-based DIF test. Possible values for functional are, for instance, "DM" for the *d* ouble-*m* aximum statistic or "LMuo" for the *u* n*o* rdered *L* agrange *M* ultiplier statistic, the *max* imally-selected *LM* test across the levels of an *o* rdered covariate (denoted "maxLMo"), or the *max* imally-selected *LM* test for a continuous covariate (denoted "maxLM").

So far, we discussed some wrapper functions for fitting IRT models that are designed to be easy to use. The underlying *mirt* package provides the following, more flexible functions:

- mirt, multipleGroup: These functions are basic fitting functions for a wide range of unidimensional and multidimensional IRT models for dichotomous and polytomous items. The item parameter estimation is based on the MML approach. In the following, we assume that the EM algorithm (Bock & Aitkin, 1981; Baker & Kim, 2004) was employed to estimate the item parameters within the MML approach. When mirt is used for estimating the item parameters, the person parameter distribution is assumed to be identical for all subgroups of the population of respondents, i.e., the standard normal distribution. multipleGroup implements the multiple group estimation approach of Bock and Zimowski (1997) to allow the modeling of different person parameter distributions in various predefined groups of respondents. mirt requires at least three arguments. The first argument, data, is the response matrix. The second argument, model, typically indicates the number of latent dimensions, which is 1 for unidimensional models. The third argument, itemtype, indicates the type of estimated IRT model and defaults to "2PL". Other options are, for instance, "Rasch", "3PL" or "4PL". multipleGroup additionally uses a group argument, which accepts a vector that defines a group membership for each respondent and allows to model impact effects between the different groups.

The following important function is used in the background:

- estfun: Methods for this generic function (which is part of the *sandwich* package, see Zeileis 2004, 2006) are contained in *mirt* as well as *psychotools* and allow for the calculation of the individual score contributions. Here, their single argument is a model object corresponding to an estimated IRT model, e.g., the return value of mirt or raschmodel. Given the importance of the scores for the underlying statistical tests, they can be seen as a central building block of our software implementation. However, it is typically not necessary to use these functions explicitly during an analysis, as will be shown in the following tutorials.

Table 2 summarizes the main R functions needed to conduct score-based tests of measurement invariance with their most important arguments.

**Table 2:** Overview of main R functions to conduct score-based tests

Function (R package)	Purpose	Central arguments	Return value
Model Fitting			
raschmodel (*psychotools*)	fitting the binary Rasch model (CML)	y (response matrix)	object of class “raschmodel”
rsmodel (*psychotools*)	fitting the rating scale model (CML)	y (response matrix)	object of class “rsmodel”
pcmodel (*psychotools*)	fitting the partial credit model (CML)	y (response matrix)	object of class “pcmodel”
plmodel (*psychotools*)	fitting the 1PL, 2PL, 3PL, 3PLu, or 4PL model (MML, *mirt* wrapper)	y (response matrix), type (type of IRT model)	object of class “plmodel”
gpcmodel (*psychotools*)	fitting the GPCM (MML, *mirt* wrapper)	y (response matrix)	object of class “gpcmodel”
mirt (*mirt*)	fitting various IRT models (MML)	data (response matrix), model (either indicating the number of latent dimensions or loadings, constraints etc. of the model), itemtype (type of IRT model, can be specified itemwise)	object of class “SingleGroupClass”
multipleGroup (*mirt*)	fitting various multiple group IRT models (MML)	as in mirt above and additionally group (defines a group membership for each respondent)	object of class “MultipleGroupClass”
Calculation of *p*-values			
sctest (*strucchange*)	generic function for calculating a test statistic and the associated *p*-value. Dispatches sctest.default by default	x (model object), order.by (typically a vector of person covariates), functional (typically a string specifying the test statistic, see main text for examples)	object of class “htest”
Background function			
estfun (*sandwich*)	generic function for calculating the individual score contributions (methods are in *mirt* and *psychotools*)	x (model object)	matrix of the individual score contributions

## Illustrations with empirical data

In our first example, we investigate DIF for a Rasch model (Rasch, 1960) with regard to a continuous and a categorical covariate, and in the second example, for a GPCM (Muraki, 1992) with regard to an ordinal and a categorical variable. More detailed simulation studies are reported in earlier studies on score-based measurement invariance tests for different other IRT models (Debelak & Strobl, 2019; Komboz et al., 2018; Strobl et al., 2011; Strobl et al., 2015; Wang et al., 2018).

Before we begin our analysis, we load the three R packages *mirt*, *psychotools* and *strucchange* that are used in these examples. Before the first usage of these packages, it is necessary to install them. This can be done by using install.packages(c("mirt", "psychotools", "strucchange")). After their installation, these packages can be loaded via:


> library("mirt")> library("psychotools")> library("strucchange")

After these packages have been loaded, we are ready to start our DIF analysis.

### Investigating DIF for a continuous or categorical covariate for the rasch model: the verbal aggression dataset

The dataset that we are going to analyze in this subsection is the Verbal Aggression dataset. These data were thoroughly discussed in the book of De Boeck and Wilson (2004) and in the paper of De Boeck et al., (2011). In these studies, Rasch models were used to model this dataset, with item parameters estimated via MML. Another reason for selecting this dataset is that it is freely available in the *psychotools* package. We therefore load this dataset first:


> data("VerbalAggression",+ package = "psychotools")

This dataset contains responses from a questionnaire assessing verbal aggressive behavior. There are overall 24 items, which were worked on by 316 respondents (243 female). For details on this questionnaire, we refer to De Boeck and Wilson (2004) or De Boeck et al., (2011). Like De Boeck et al., (2011), we code the response “no” as 0 and all other responses as 1. Person covariates that are part of this dataset are gender and a score for anger. In the context of this paper, we are interested in testing for DIF with respect to the anger and gender variables.

VerbalAggression$resp2 contains the responses of all 314 respondents (as rows) to the 24 items (as columns), coded by 0 and 1, VerbalAggression$anger contains the anger scores for all respondents. VerbalAggression$gender provides information on the respondents’ gender, using the categories female and male. We start our analysis by selecting an IRT model and an estimation approach for our analysis. Like De Boeck and Wilson (2004) and De Boeck et al., (2011), we base our analysis on the Rasch model for dichotomous data, but focus on the first six items, which correspond to the responses to the first frustrating situation in the test (“A bus fails to stop for me.”). These six items use three behaviors (“cursing”, “scolding”, “shouting”) in the “doing” and “wanting” mode as a possible reaction to this situation. As an estimation approach, we choose CML estimation, since this method makes less strict assumptions about the distribution of the person parameters. We therefore use the *psychotools* package for estimating the item parameters. The following R command fits the Rasch model and stores the results in the raschmodel object RM_VA:


> RM_VA <- raschmodel(+ VerbalAggression$resp2[, 1:6])

The following command carries out a score-based test with regard to the covariate anger. The order.by argument contains the covariate that should be used for DIF testing. As a test statistic, we use the maximum LM test, which corresponds to the argument maxLM:


> sctest(RM_VA, order.by =+ VerbalAggression$anger,+ functional = "maxLM")


M-fluctuation testdata: RM_VA f(efp) = 14.019, p-value =0.2308

sctest returns the value of the test statistic as well as the associated *p*-value. As the result shows, there is no significant DIF effect with regard to anger.

We also apply a score-based test which tests the null hypothesis of measurement invariance with regard to gender. In the following, we set the argument functional to LMuo (i.e., we use the unordered LM test) to account for the nominal scale of our person covariate gender. This leads to:


> sctest(RM_VA,+ order.by = VerbalAggression$gender,+ functional = "LMuo")


M-fluctuation testdata: RM_VA f(efp) = 9.8788, p-value =0.07874

This result does not indicate a significant DIF effect either.

### Investigating DIF for an ordinal or categorical covariate for the generalized partial credit model: the generic conspiracist beliefs dataset

Our second example dataset is also available in the *psychotools* package and contains the responses of 2449 persons to 15 items measuring belief in conspiracy theories as well as two covariates, area (area one lived in as a child) and gender. The area covariate is of ordinal nature and takes on the values “rural”, “suburban” and “urban”.

As in the first example, we first select an IRT model for our analysis. Since our items are polytomous and our dataset is large enough to allow a sufficiently accurate estimation of slope parameters, we choose the GPCM. As this model does not belong to the family of Rasch-type models, we rely on MML estimation for estimating the parameters. A third decision concerns the use of a wrapper function for fitting the model. This decision does not affect the results of the analysis, but the wrapper functions included in the *psychotools* package are generally more convenient in their application. First, we load the dataset from the *psychotools* package:


> data("ConspiracistBeliefs2016",+ package = "psychotools")

The responses are named resp and the variable for which impact is modeled is area. Both are stored in the data frame ConspiracistBeliefs2016. The following code estimates the GPCM item parameters while accounting for a possible impact effect with regard to area; we remind the reader that an impact effect corresponds to a shift in the distribution of the ability parameters. As was already argued in the introduction, this modeling choice avoids a possibly increased Type I error rate of the following score-based test. The model fitting is done with the gpcmodel function, which is one of the wrapper functions. We start our analysis by fitting the GPCM, which is done using *mirt* in the background. The impact argument specifies the variable for which impact is modeled. The maxit argument determines the maximum number of EM iterations for this estimation procedure. The default value in *mirt* (and analogously in the wrappers in *psychotools*) is 500, but larger datasets, as the one analyzed here, typically require a larger number of iterations. If the number of iterations is too low and the estimation algorithm does not converge, a warning will be printed.


> GPCM_Area <- gpcmodel(+ ConspiracistBeliefs2016$resp,+ impact = ConspiracistBeliefs2016$area,+ maxit = 1000)

Based on the fitted model, a score-based test with regard to the area covariate (employing the maximally-selected LM test, maxLMo, across the levels of the ordered covariate) can be carried out via:


> sctest(GPCM_Area,+ order.by = ConspiracistBeliefs2016$area,+ functional = "maxLMo")


M-fluctuation testdata: GPCM_Area f(efp) = 89.605, p-value =0.2205

This test is not significant. Therefore, we do not find a significant DIF effect with regard to the area covariate. It should be noted that we also could have proceeded to only test for DIF in individual items. In our example that uses the GPCM whose item parameters were estimated by MML estimation, this can be achieved by identifying the respective item parameters and providing their indices via the parm argument of sctest. For instance, if we wanted to test for DIF in only the first item, and therefore in the first five item parameters (that is, one slope and four threshold parameter), this could be done via:


> sctest(GPCM_Area,+ order.by = ConspiracistBeliefs2016$area,+ functional = "maxLMo", pars = 1:5)


M-fluctuation testdata: GPCM_Area f(efp) = 89.605, p-value= 0.2206

The result is again not significant, which indicates an absence of DIF in the first five item parameters, which corresponds to the parameters of the first item. On a statistical level, this corresponds to a DIF test that checks whether the item parameters of the first item are invariant with regard to the area covariate while assuming that the remaining item parameters are stable. Please note that the order of the item parameters in the pars argument depends on the estimated model. It is equal to the order in the output of the estfun function, which can be directly inspected by the user. In practical applications of these item-wise DIF tests, the assumption that the parameters of all other items are stable should be critically reflected by the user before interpreting the resulting *p*-value. In the DIF testing literature, items that are set equal between the groups are termed anchor items, and it is important to select these items in a sensible way.

At this point, we also want to point to the anchortest function available in the *psychotools* package, which allows users to conduct an itemwise Wald test for pre-defined groups with “built-in” anchor selection based on a variety of methods. The anchor selection methods available in this function are described in Kopf et al. (2015a, 2015b).

For completeness, we also test for DIF regarding the covariate gender. First, we fit the GPCM modeling potential impact in gender:


> GPCM_Gender <- gpcmodel(+ ConspiracistBeliefs2016$resp,+ impact = ConspiracistBeliefs2016$gender,+ maxit = 1000)

Second, we conduct the score-based test for all items with the unordered Lagrange multiplier statistic, using the functional LMuo:


> sctest(GPCM_Gender,+ order.by =+ ConspiracistBeliefs2016$gender,+ functional = "LMuo")


M-fluctuation testdata: GPCM_Gender f(efp) = 288.61, p-value= 7.993e-11

This test finds a *p*-value smaller than 0.001. We therefore reject the null hypothesis of measurement invariance with regard to gender. We conclude that at least one item parameter differs between females and males.

## Discussion

In this tutorial, we provided a detailed introduction to the theory of score-based tests for detecting DIF in IRT models, as well as their application within the R system for statistical computing relying on the packages *psychotools*, *mirt* and *strucchange*. The underlying family of statistical tests was developed for investigating the invariance of ML estimators with regard to categorical, ordinal and continuous covariates. It can be applied to both CML and MML estimated IRT models.

New functions that we have added to the *psychotools* and *mirt* packages make these kind of score-based tests of measurement invariance accessible to the general user. Easy-to-use wrapper functions included in *psychotools* now allow for a straightforward application of these tests without requiring a detailed knowledge of the underlying R commands. So far, these wrapper functions are available for the Rasch model (with MML estimation; 1PL model), the 2PL and 3PL models of Birnbaum (1968), the more general 4PL model (Barton and Lord, 1981), as well as the GPCM (Muraki, 1992), including the partial credit model (Masters, 1982). Alternatively, users can use *mirt* directly for an application of these tests to all kinds of IRT models estimated within the MML approach. These applications might be particularly interesting for substantive researchers. Future extensions might contain additional wrapper functions, for instance for multidimensional models like the multidimensional compensatory 2PL model (Reckase, 2009). Since it is already possible to obtain MML estimates for these models with the *mirt* package, it is also possible to check their item parameter invariance with score-based measurement invariance tests, although no simulation results regarding the power of these tests have been published so far for these models. By similar means it is also possible to carry out a power analysis of score-based measurement invariance tests as part of the IRT analysis of empirical data. This application might be of interest for psychometricians who wish to investigate the psychometric characteristics of these tests. The *psychotools* package contains a vignette on how to conduct simulation studies with score-based tests.

We want to conclude this paper with a discussion of possible extensions of the presented method. In principle, these extensions could either address situations where assumptions underlying the methods presented here are violated, or they could extend the functionality of the software.

We start with extensions of the first type. This paper considers a scenario where all respondents worked 1) on the same item set and 2) a likelihood-based approach is used for estimating the item parameters. This scenario is fairly general and covers a wide range of situations encountered in empirical data analysis. A first extension of practical interest is the development of analogous methods for Bayesian estimation methods, which are also commonly used in IRT analysis (Fox, 2010; Levy & Mislevy, 2016). A second extension could be a scenario where not all respondents worked on the same item set. A typical example is the application of a computerized adaptive test or a multistage test (Van der Linden & Glas, 2010; Yan et al., 2016). These techniques of item presentation are well-known in educational settings and have in common that the selection of items worked on during a test (partly) depends on previous test behavior, whereas the item parameters are typically treated as known.

We also mention some possible extensions to the functionality of the software. An example concerns the development of tree-based methods based on recursive model-based partitioning. This type of method is based on simultaneous tests for measurement invariance with regard to multiple person covariates. It aims at a data-driven detection of groups for which no significant DIF effects can be detected. For Rasch-type models and the CML approach, such methods are already provided in the *psychotree* package (Komboz et al., 2018; Strobl et al., 2015).

Another field for future work could be the inclusion and evaluation of sensitivity statistics. These statistics indicate the expected change of model parameters if they are no longer assumed to be invariant across groups of respondents. After score-based tests have detected a violation of invariance, statistics of this kind could help to investigate the practical relevance of this model violation. An example is the expected parameter change (EPC)-interest statistic, which was suggested by Oberski (2014) for structural equation models. A closely related idea is the inclusion of effect sizes for DIF effects (Chalmers, 2018; Steinberg and Thissen, 2006).
